# Production in yeast of pseudotype virus-like particles harboring functionally active antibody fragments neutralizing the cytolytic activity of vaginolysin

**DOI:** 10.1186/1475-2859-10-109

**Published:** 2011-12-15

**Authors:** Milda Pleckaityte, Aurelija Zvirbliene, Indre Sezaite, Alma Gedvilaite

**Affiliations:** 1 Vilnius University, Institute of Biotechnology, Department of Eukaryote Genetic Engineering, Graiciuno 8, LT-02241 Vilnius, Lithuania; 2 Vilnius University, Institute of Biotechnology, Department of Immunology and Cell Biology, Graiciuno 8, LT-02241 Vilnius, Lithuania

**Keywords:** Recombinant antibodies, virus-like particles, vaginolysin

## Abstract

**Background:**

Recombinant antibodies can be produced in different formats and different expression systems. Single chain variable fragments (scFvs) represent an attractive alternative to full-length antibodies and they can be easily produced in bacteria or yeast. However, the scFvs exhibit monovalent antigen-binding properties and short serum half-lives. The stability and avidity of the scFvs can be improved by their multimerization or fusion with IgG Fc domain. The aim of the current study was to investigate the possibilities to produce in yeast high-affinity scFv-Fc proteins neutralizing the cytolytic activity of vaginolysin (VLY), the main virulence factor of *Gardnerella vaginalis*.

**Results:**

The scFv protein derived from hybridoma cell line producing high-affinity neutralizing antibodies against VLY was fused with human IgG1 Fc domain. Four different variants of anti-VLY scFv-Fc fusion proteins were constructed and produced in yeast *Saccharomyces cerevisiae*. The non-tagged scFv-Fc and hexahistidine-tagged scFv-Fc proteins were found predominantly as insoluble aggregates and therefore were not suitable for further purification and activity testing. The addition of yeast α-factor signal sequence did not support secretion of anti-VLY scFv-Fc but increased the amount of its intracellular soluble form. However, the purified protein showed a weak VLY-neutralizing capability. In contrast, the fusion of anti-VLY scFv-Fc molecules with hamster polyomavirus-derived VP2 protein and its co-expression with VP1 protein resulted in an effective production of pseudotype virus-like particles (VLPs) that exhibited strong VLY-binding activity. Recombinant scFv-Fc molecules displayed on the surface of VLPs neutralized VLY-mediated lysis of human erythrocytes and HeLa cells with high potency comparable to that of full-length antibody.

**Conclusions:**

Recombinant scFv-Fc proteins were expressed in yeast with low efficiency. New approach to display the scFv-Fc molecules on the surface of pseudotype VLPs was successful and allowed generation of multivalent scFv-Fc proteins with high VLY-neutralizing potency. Our study demonstrated for the first time that large recombinant antibody molecule fused with hamster polyomavirus VP2 protein and co-expressed with VP1 protein in the form of pseudotype VLPs was properly folded and exhibited strong antigen-binding activity. The current study broadens the potential of recombinant VLPs as a highly efficient carrier for functionally active complex proteins.

## Background

Recombinant antibodies are widely used in therapeutic, diagnostic and research settings. Different variants of recombinant antibodies have been described to date. Chimeric and humanized antibodies represent important biopharmaceutical products for the immunotherapy of malignant and inflammatory diseases [1]. The advantage of full-length recombinant immunoglobulin molecule is its ability to perform both antigen-binding and effectors' functions. For some applications, functionally active recombinant antibody fragments instead of full-length antibodies can be used. Single chain variable fragments (scFvs) remain attractive recombinant molecules because of their selection *in vitro *approaches, lack of glycosylation, small size and tissue penetration efficacy, lower immunogenicity as a result of elimination of constant domains of the antibody, easier and less costly manufacture [2,3]. The scFv consists of variable regions of light (VL) and heavy (VH) immunoglobulin chains forming antigen-binding domains engineered into a single polypeptide [4]. VL and VH regions are usually joined by a flexible linker sequence. The scFvs are mainly produced as monomeric structures displaying monovalent antigen-binding activity. However, the lack of Fc domain impairs the stability of the scFv molecule. As a consequence, the scFvs are rapidly degraded in serum and have short circulating half-lives [5]. Several strategies have been used to circumvent the drawbacks of scFvs and obtain better clearance properties. Further engineering allowed forming of multivalent antibody fragments (diabodies, triabodies) with single or multiple specificities to different target antigens [6]. An alternative approach includes scFv fusion with IgG Fc domain leading into IgG-like format [7-9]. In addition, the scFv being a monomer molecule after the fusion with Fc regains the avidity because of dimerization [9]. Taken together, scFv-Fc fusion protein retains the affinity and specificity of the parent scFv along with the prolonged serum half-life and bivalent binding [7].

Recombinant full-length immunoglobulins are usually produced in eukaryote cells. Mammalian expression systems ensure proper folding and post-translational modification of recombinant antibodies. However, the main disadvantages of cell cultures are low expression levels, expensive and time-consuming production of recombinant proteins [10]. The employment of yeast and plant expression systems for the generation of humanized recombinant antibodies has also been demonstrated [11-15]. For the production of antibody fragments (scFv, Fab fragments, diabodies) yeast and bacterial cells are widely used because recombinant antibody fragments do not require glycosylation for their biological activities and are relatively easily assembled [16]. However, often introduction of different modifications in yeast or *E. coli *cells is necessary to optimize the expression of antibody fragments. For example, remarkably increased production of scFv in *Saccharomyces cerevisiae *was obtained when two chaperones were overexpressed together with scFv and yeast growth temperature was reduced [17]. An alternative approach to overcome aggregation leading to subsequent degradation of scFv expressed in *S. cerevisiae *may be the presentation of scFv molecules on the surface of virus-like particles (VLPs) as we demonstrated in the current study.

Recently, we have developed neutralizing monoclonal antibodies (MAbs) against the protein toxin vaginolysin (VLY), the main virulence factor of *Gardnerella vaginalis *[18]. VLY belongs to the cholesterol-dependent cytolysins (CDCs), a family of pore-forming toxins [19]. These toxins cause lysis of cellular membrane and are thought to play a key role in the virulence of bacteria [20]. The MAbs against VLY were shown to bind the toxin with high affinity and inhibit VLY-mediated hemolysis of human erythrocytes [18]. Inhibition of cytolytic activity of VLY may have important physiologic relevance, as the lysis of vaginal epithelial cells by VLY is considered to be a key step in progression of bacterial vaginosis and predispose to the disease-associated complications [21].

In the present study, the scFv derived from hybridoma cell line producing high-affinity neutralizing MAbs against VLY [18] was fused with human IgG1 Fc domain comprising the CH2 and CH3 domains and the hinge region [9]. We have expressed the anti-VLY scFv-Fc construct in yeast *S. cerevisiae *in four different formats and demonstrated that pseudotype VLPs consisting of hamster polyomavirus (HaPyV)-derived capsid proteins represented highly efficient carrier for a functionally active scFv-Fc molecules with VLY-neutralizing activity.

## Results

### Expression of anti-VLY scFv-Fc constructs in yeast

**Figure 1:**
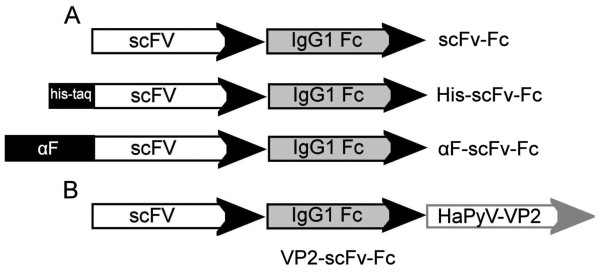
**Schematic representation of anti-VLY scFv-Fc constructs expressed in yeast**.

For the production of anti-VLY scFv fused to a human IgG1 Fc domain in yeast, the expression plasmids encoding different scFv-Fc variants (Figure 1A) were constructed: (i) non-tagged scFv-Fc, (ii) scFv-Fc harboring the hexahistidine tag (His-tag) at the N-terminus (His-scFv-Fc), (iii) scFv-Fc harboring yeast α-factor signal sequence at the N-terminus (αF-scFv-Fc). The respective DNA sequences were cloned into yeast expression vector pFX7 [22] and transformed into yeast *S. cerevisiae*. The addition of His-tag sequence was considered to be useful for further purification of recombinant scFv-Fc protein. Yeast α-factor signal sequence was added in order to direct the secretion of anti-VLY scFv-Fc to the growth medium that may significantly simplify purification procedure of the recombinant protein.

The production of target proteins was subjected under the control of an inducible galactose promoter. The expression of all recombinant anti-VLY scFv-Fc constructs was analyzed in yeast strain AH22-214 and its derivative AH22-214p lacking peptidase Pep4 both by gel electrophoresis (SDS-PAGE) and Western blot using anti-human IgG conjugated to horse-radish peroxidase (HRP). The production of recombinant anti-VLY scFv-Fc proteins were detected only in yeast strain AH22-214p. No expression of the anti-VLY scFv-Fc constructs in yeast strain AH22-214 cells was detected (data not shown).

**Figure 2:**
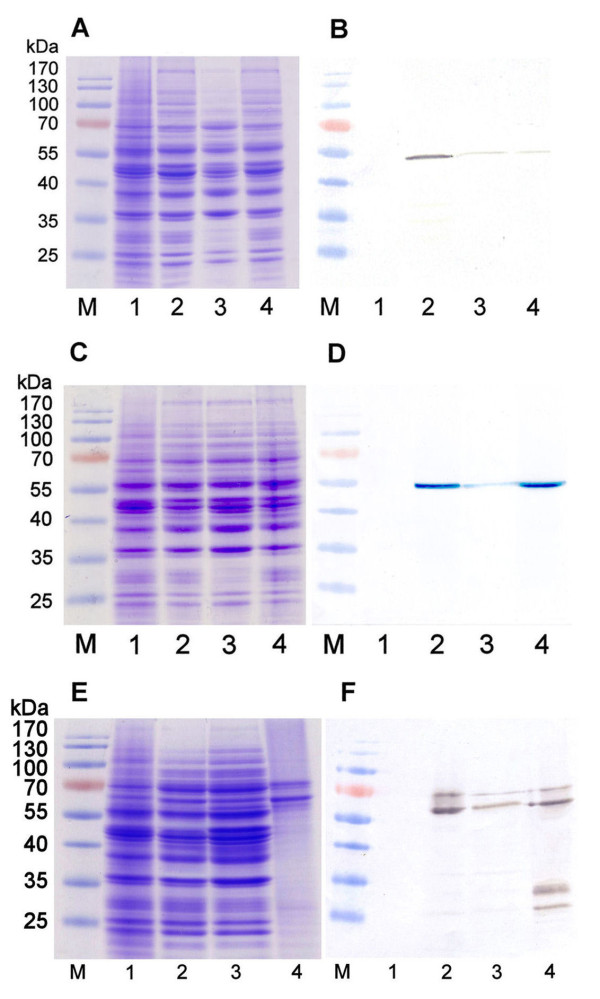
**Analysis of the production of anti-VLY scFv-Fv, His-scFv-Fv and αF-scFv-Fv proteins in yeast strain AH22-214p**. (A, C, E): Coomassie blue-stained SDS-PAGE; (B, D, F): Western blot with HRP-labeled secondary antibody against human IgG. (A, B): In lanes: 2-whole crude lysate of yeast transformed with pFX7-scFv-Fc plasmid, 3-the soluble fraction recovered after centrifugation of whole crude lysate of yeast transformed with pFX7-scFv-Fc plasmid, 4-pellet recovered after centrifugation of whole crude lysate of yeast transformed with pFX7-scFv-Fc plasmid. (C, D): In lanes: 2-whole crude lysate of yeast transformed with pFX7-His-scFv-Fc plasmid, 3-the soluble fraction recovered after centrifugation of crude lysate of yeast transformed with pFX7-His-scFv-Fc plasmid, 4-pellet recovered after centrifugation of crude lysate of yeast transformed with pFX7-His-scFv-Fc plasmid. (E, F): In lanes: 2-whole crude lysate of yeast transformed with pFX7-αF-scFv-Fc plasmid, 3-the soluble fraction recovered after centrifugation of crude lysate of yeast transformed with pFX7-αF-scFv-Fc plasmid, 4-αF-scFv-Fc purified using protein A Sepharose (lane 4). In all gels negative control sample of whole crude lysate of *S. cerevisiae *cells, transformed with empty vector pFX7 was loaded on lane 1 and pre-stained protein weight marker (Thermo Scientific Fermentas) was loaded on lane M.

The non-tagged anti-VLY scFv-Fc fusion protein (molecular mass of approximately 51 kDa) was not visible in SDS-PAGE (Figure 2A) but was detected with HRP-labeled anti-human IgG as a faint a band in the whole crude lysate of transformed yeast strain AH22-214p cells (Figure 2B, lane 2) and its soluble and insoluble fractions (Figure 2B, lanes 3, 4). However, the expression level of the soluble anti-VLY scFv-Fc protein in yeast strain AH22-214p was too low for further purification and functional studies. The obtained results demonstrated low efficiency of yeast expression system for the production of the non-tagged anti-VLY scFv-Fc construct.

The addition of His-tag sequence to the N-terminus of anti-VLY scFv-Fc construct resulted in higher expression level as compared to the non-tagged protein. The His-scFv-Fc protein was detected both by SDS-PAGE and Western blot in the whole crude lysate of transformed yeast AH22-214p cells (Figure 2C, D, lane 2). However, the main part of His-scFv-Fc was found in the insoluble fraction of yeast cell lysate (Figure 2C, D, lane 4). The level of anti-VLY His-scFv-Fc in the soluble fraction of yeast cell lysate was considered as insufficient for further purification and activity testing.

It was expected that yeast α-factor signal sequence added to the N-terminus of scFv-Fc will assist the secretion of recombinant protein αF-scFv-Fc into yeast growth medium. However, the αF-scFv-Fc protein was not detected in yeast growth medium analyzed both by SDS-PAGE and Western blot (data not shown). Nevertheless, addition of yeast α-factor signal sequence improved the intracellular production of anti-VLY scFv-Fc. A protein band of approximately 60 kDa corresponding to the αF-scFv-Fc was found in the soluble fraction of yeast cell lysate examined both by SDS-PAGE and Western blot (Figure 2E, F, lane 3). The amount of soluble αF-scFv-Fc protein was sufficient for purification and further activity testing. The soluble fraction of yeast cell lysate was collected and anti-VLY αF-scFv-Fc protein was purified using affinity chromatography on protein A which binds the Fc domain of the target protein (Figure 2E, F, lane 4). Western blot analysis of unpurified as well as purified anti-VLY αF-scFv-Fc protein revealed two immunostained bands of approximately 60 and 70 kDa (Figure 2F, lane 4). The principal band of 60 kDa represents anti-VLY αF-scFv-Fc protein with unprocessed yeast α-factor signal sequence at the N-terminus. The protein fraction of 70 kDa most likely represents glycosylated form of the αF-scFv-Fc protein with unprocessed yeast α-factor signal sequence at the N-terminus. Although the anti-VLY αF-scFv-Fc protein demonstrated better stability than non-tagged scFv-Fc or His-scFv-Fc proteins, the immunoreactive 30 and 25 kDa bands detected by Western blot in the purified protein preparation may evidence the degradation products. These data confirmed the tendency of yeast-expressed scFv-Fc proteins to degradation.

### Expression of anti-VLY scFv-Fc construct displayed on the surface of pseudotype VLPs

**Figure 3:**
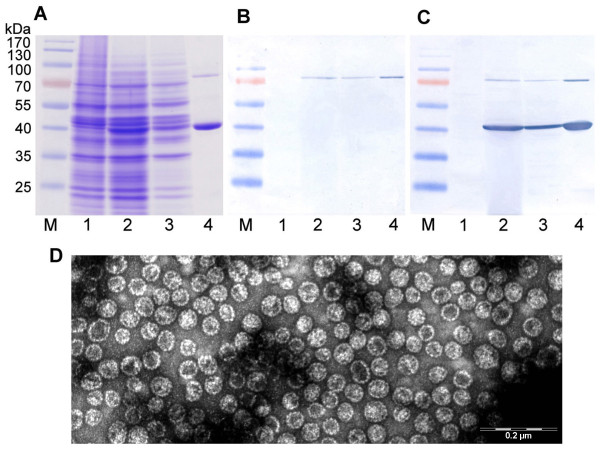
**Analysis of the expression of anti-VLY VP1/VP2-scFv-Fv pseudotype VLPs in yeast**. (A): Coomassie blue-stained SDS-PAGE; (B): Western blot with HRP-conjugated anti- human IgG; (C): Western blot with mouse polyclonal antibodies against VP1/VP2 VLPs. The same samples were run on each gel. In lanes: 1-negative control sample from whole crude lysate of *S. cerevisiae *cells, transformed with empty vector pFX7; 2-whole crude lysate of yeast transformed with pFGG3-VP1/VP2-scFv-Fc plasmid, 3-the soluble fraction recovered after centrifugation of crude lysate of yeast transformed with pFGG3-VP1/VP2-scFv-Fc plasmid, 4-VLPs consisting of VP1 protein and fusion protein VP2-scFv-Fc purified using sucrose and CsCl gradients; M-pre-stained protein weight marker (Thermo Scientific Fermentas). (D): Electron microscopy pictures of VP1/VP2-scFv-Fv pseudotype VLPs, stained with 2% aqueous uranyl acetate solution and examined by Morgagni 268 electron microscope.

In order to improve the expression level of anti-VLY scFv-Fc in yeast, as well as to reduce its instability and insolubility a new approach was applied. The gene encoding anti-VLY scFv-Fc protein was fused with gene encoding modified HaPyV-derived minor capsid protein VP2 and co-expressed in yeast strain AH22-214p together with HaPyV gene encoding the major capsid protein VP1 (Figure 1B). The production of both VP1 and VP2-scFv-Fc proteins was induced by adding galactose. After induction of the synthesis of VP1 and VP2-scFv-Fc proteins the lysate of harvested yeast cells was examined both by SDS-PAGE and Western blot. The analysis of yeast cell lysate by SDS-PAGE revealed a protein band of approximately 42 kDa that was identified as an intact VP1 protein (Figure 3A, lanes 2, 3). In the Western blot a protein band of approximately 80 kDa corresponding to the molecular mass of anti-VLY VP2-scFv-Fc fusion protein was detected (Figure 3B, lanes 2, 3). Polyclonal antibodies against HaPyV VP1 and VP2 proteins immunostained the same 80 kDa and 42 kDa bands corresponding to the VP2-scFv-Fc and VP1 proteins, respectively (Figure 3C, lanes 2, 3).

The VP1/VP2-scFv-Fc proteins generated in yeast were successfully purified by the method adapted for the purification of VLPs using sucrose and CsCl density centrifugation. The analysis of purified VP1/VP2-scFv-Fc proteins by both SDS-PAGE and Western blot revealed two expected homogenous protein bands (Figure 3A, B, C, lane 4). The purified VP1/VP2-scFv-Fc proteins were negatively stained and subjected to electron microscopy examination. It was determined that the VP1/VP2-scFv-Fc proteins efficiently self-assembled into so-called "pseudotype" VLPs (consisting of intact VP1 and modified VP2 viral proteins) similar in their size and shape to unmodified HaPyV-VP1 VLPs. The diameter of recombinant pseudotype VLPs was 45-50 nm, which is typical for polyomavirus capsid (Figure 3D). It was calculated that approximately 19 scFv-Fc molecules were displayed on one pseudotype VLP.

### Neutralizing activity of the αF-scFv-Fc protein and pseudotype VLPs harboring scFv-Fc

Antigen-binding activity of anti-VLY αF-scFv-Fc protein eluted from protein A-Sepharose column as well as purified pseudotype VLPs harboring scFv-Fc was demonstrated by an indirect ELISA using both anti-human IgG and anti-mouse IgG secondary antibody (data not shown). The ability of the anti-VLY αF-scFv-Fc protein purified by affinity chromatography and scFv-Fc displayed on the surface of recombinant VP1/VP2-scFv-Fc pseudotype VLPs to inhibit the hemolytic activity of VLY was assayed *in vitro *using human erythrocyte suspension.

**Figure 4:**
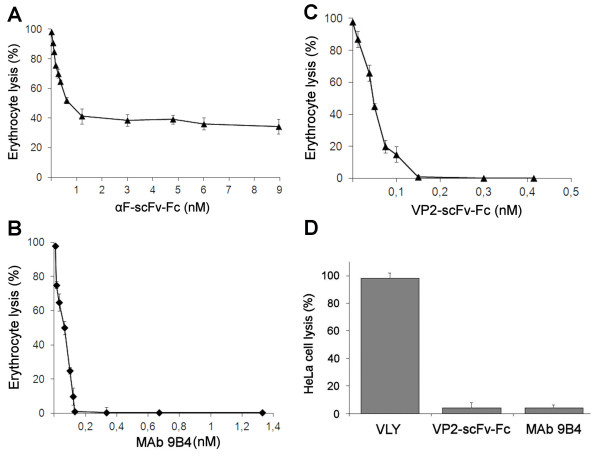
**Inhibition of VLY-mediated cytolysis by anti-VLY αF-scFv-Fc, mAb 9B4 and pseudotype VP1/VP2-scFv-Fc VLPs**. (A, B, C): The inhibition of VLY-mediated lysis of human erythrocytes (% of hemolysis cells) was evaluated after addition of human erythrocyte suspension to VLY (5 ng/ml) pre-incubated with (A): αF-scFv-Fc protein at concentrations ranging from 3.6 ng/ml (6 × 10^-11 ^M) to 540 ng/ml (8.96 x10^-9 ^M); (B): MAb 9B4 at concentrations ranging from 1 ng/ml (6.7 × 10^-11 ^M) to 200 ng/ml (1.33 × 10^-9 ^M); (C): VP1/VP2-scFv-Fc VLPs at concentrations of VP2-scFv-Fc protein ranging from 1 ng/ml (1.25 × 10^-11 ^M) to 33 ng/ml (4.1 × 10^-10 ^M). (D): The inhibition of VLY-mediated lysis of human cervical epithelial HeLa cells was evaluated after their exposure to VLY alone (3 μg/ml) or VLY (3 μg/ml) preincubated either with VP1/VP2-scFv-Fc VLPs (VP2-scFv-Fc concentration 3 μg/ml or 3.8 × 10^-8 ^M) or MAb 9B4 (50 μg/ml or 3.33 x10^-7 ^M). Cell viability was determined by colorimetric assay using MTS staining. Values were normalized to 100% for each assay.

Anti-VLY αF-scFv-Fc protein was used at the concentrations ranging from 3.6 ng/mL (6 × 10^-11 ^M) to 0.54 μg/mL (8.96 × 10^-9 ^M) and the recombinant VLY was used at the concentration of 5 ng/mL (8.5 × 10^-11 ^M) that was proven to be sufficient for a complete lysis of human erythrocytes [18]. However, anti-VLY αF-scFv-Fc protein induced only partial neutralization of the VLY activity. Even at high concentrations of the αF-scFv-Fc protein (540 ng/mL or 8.96 × 10^-9 ^M) its inhibitory effect did not exceed 60% (Figure 4A) of that observed with 20 ng/mL (1.33 × 10^-10 ^M) of full-length parental antibody clone 9B4 (Figure 4B). Most probably, the incomplete neutralization of the VLY activity was caused by a lower affinity of the monovalent αF-scFv-Fc construct as compared to the full-length antibody.

In contrast, the scFv-Fc molecule fused with VP2 protein and presented on the surface of pseudotype VP1/VP2 VLPs was functionally active and inhibited the cytolytic activity of VLY with high efficiency. Complete neutralization of VLY in the *in vitro *hemolytic assay was observed at concentrations of chimeric VP2-scFv-Fc protein exceeding 12 ng/mL or 1.5 × 10^-10 ^M (Figure 4C). Thus, the VLY-neutralizing potency of VP1/VP2-scFv-Fc was comparable to that obtained with 20 ng/mL or 1.33 × 10^-10 ^M of full-length parental MAb 9B4 (Figure 4B).

The neutralizing activity of anti-VLY scFv-Fc displayed on the surface of recombinant VP1/VP2-scFv-Fc pseudotype VLPs was also tested in additional *in vitro *cytolytic assay using human cervical epithelial HeLa cell line and demonstrated high VLY-neutralizing potency of anti-VLY VP1/VP2-scFv-Fc VLPs comparable to that of full-length MAb 9B4. The extent of the VLY-mediated lysis of HeLa cells was evaluated by colorimetric MTS assay. It was determined that 3 μg/mL (3.8 × 10^-8 ^M) of the chimeric VP2-scFv-Fc protein displayed on VP1/VP2-scFv-Fc VLPs induced complete neutralization of the VLY-mediated cytolysis as effective as 50 μg/mL or 3.3 × 10^-7 ^M full-length anti-VLY antibody 9B4 (Figure 4D).

## Discussion

Yeast is attractive host for production of heterologous proteins [23]. The main advantages of the production of recombinant antibodies in yeast are the possibilities to use cheap, defined growth media and generate high cell densities in short cultivation times [24]. Previous studies show that scFv-Fc proteins assemble in yeast easier when compared to bacterial expression systems [7,25-27]. In line with these data, our attempts to express anti-VLY scFv-Fc construct in *E. coli *were unsuccessful-the scFv-Fc protein was found to be insoluble and the efforts for its refolding were inefficient (data not shown). Therefore, in the current study yeast expression system has been selected for the synthesis of anti-VLY scFv-Fc protein. To obtain functionally active scFv-Fc protein in yeast, four scFv-Fc constructs were generated: non-tagged scFv-Fc, His-scFv-Fc, αF-scFv-Fc and VP1/VP2-scFv-Fc assembled into pseudotype VLPs (Figure 1). As the anti-VLY scFv-Fc constructs were not detectable after induction in yeast strain AH22-214 containing an intact yeast peptidase Pep4, we hypothesize that these constructs were highly sensitive to proteolysis. In contrast, the expression of all four constructs was obtained in the yeast strain AH22-214p where Pep4 peptidase was deleted. Despite different expression levels of non-tagged scFv-Fc, His-scFv-Fc and αF-scFv-Fc, a major part of all these proteins was found as insoluble aggregates what could be associated with protein misfolding (Figure 2). It is considered that the exposure of the hydrophobic surfaces on the VL and VH chains of scFv plays an important role in its aggregation in the yeast cell [28]. The hydrophobic regions of the scFv molecule responsible for keeping the variable regions of the heavy and light chains together could also interact with other molecules in the cell. The aggregation of scFv molecules in *S. cerevisiae *may also result in their subsequent degradation because of induction of the unfolded protein response [29,30]. In agreement with these reports, our attempts to express in yeast the non-tagged scFv-Fc protein were unsuccessful. Although the expression of the scFv-Fc was improved after adding the His-tag at its N-terminus, the amount of the soluble form of His-scFv-Fc protein remained too low for further processing. It was anticipated that the addition of yeast α-factor signal sequence will support the secretion of the soluble form of scFv-Fc protein. However, despite the fact that the part of the αF-scFv-Fc protein was found in the soluble cell lysate (Figure 2F, lanes 2, 3, 4), the recombinant protein was not detected in yeast growth medium. Most likely the secretion of the αF-scFv-Fc protein was hampered by its improper folding and aggregates were formed in the ER and vacuolar-like organelles as demonstrated for other recombinant proteins in yeast [28]. Surprisingly, the intracellular production of the αF-scFv-Fc protein was improved and the amount of its soluble form was sufficient for purification. Although yeast α-factor signal sequence stabilized recombinant αF-scFv-Fc protein, it also might interfere with the scFv fragment and reduce the affinity of scFv-Fc to its target. Another reason why the αF-scFv-Fc protein demonstrated a weak VLY-neutralizing activity as compared to the full-length antibody 9B4 could be the monovalent structure or irregularly aggregated form of the purified αF-scFv-Fc protein.

To improve the stability and binding properties of anti-VLY scFv-Fc protein we have applied a new approach for its expression in yeast. The scFv-Fc molecule was fused with HaPyV-derived minor capsid protein VP2 and expressed on the surface of HaPyV-derived pseudotype VLPs consisting of capsid proteins VP1 and VP2. Polyomaviruses are nonenveloped viruses with an icosahedral capsid, approximately 45 nm in diameter. As the crystal structures of the virions of SV-40 [31] and murine polyomavirus [32] have been resolved, it is known that the capsid of polyomaviruses mainly consists of 72 pentamers formed by 360 copies of the VP1 protein. One minor capsid protein, either VP2 or VP3, binds in the central 5-fold cavity of each VP1 pentamer [33]. VLPs can be efficiently produced by heterologous expression of one or more viral structural proteins which spontaneously self-assemble into structures usually similar to the authentic viruses they are derived from, but non-infectious because they are free of viral genetic material [34]. The capability of recombinant polyomavirus-derived capsid proteins to form VLPs has been demonstrated in earlier reports [35]. Our previous studies show that HaPyV-derived VLPs represent a useful tool for protein engineering. HaPyV VP1 VLPs are powerful vehicles for the foreign peptides of interest as they tolerate inserts of different size and origin (from 9 to 120 amino acid (aa)-long) at certain VP1 sites [36-38]. The assembly capacity of HaPyV VP1 protein and its interaction with the VP2 protein which was fused to the anti-VLY scFv-Fc sequence was exploited in this study for the production of pseudotype VLPs exhibiting VLY-binding activity.

It was known that the C-terminal part of VP2 protein was necessary for interaction with VP1 pentamer [33] therefore, VP2 N-terminus was used to join the scFv-Fc molecule. For this purpose, 100 aa from N-terminus of VP2 protein was deleted and replaced with anti-VLY scFv-Fc expecting that recombinant antibody fragments will be displayed on the surface of VLPs. Subsequent purification of recombinant proteins by density ultracentrifugation adapted for VLP purification and electron microscopy analysis confirmed that pseudotype VP1/VP2-scFv-Fc VLPs were efficiently produced in yeast *S. cerevisiae*. As maximum 72 minor capsid proteins, either VP2 or VP3 could be included into VP1/VP2 VLP [33] consequently up to 72 chimeric VP2-scFv-Fc molecules might be incorporated into one VLP. However, the attachment of large fusion protein on the surface of pseudotype VLP readjusted the quantity of VP2-scFv-Fc to approximately 19 molecules per VLP. The different production levels of co-expressed VP1 and VP2-scFv-Fc proteins (what is very conducive to the VP1/VP2-scFv-Fc pseudotype VPL formation) was ensured by using the pFGG3 vector [39]. The pFGG3 vector is equipped with two promoters induced to different degrees because of the shortage of Gal4 activator in yeast cells.

The incorporation of VP2-scFv-Fc into pseudotype VLPs prevented scFv-Fc misfolding and aggregation as there was no protein solubility problem during purification of VLPs. Furthermore, it was demonstrated that anti-VLY scFv-Fc molecules displayed on the surface of VLPs were stable and functionally active in all used applications. VP1/VP2-scFv-Fc VLPs inhibited VLY-mediated cell lysis with high efficiency comparable to that of full-length 9B4 antibody. High neutralizing activity of VP1/VP2-scFv-Fc could be explained by its multimeric structure and presentation of approximately 19 scFv-Fc molecules on the surface of one pseudotype VLP what regained the avidity to VLY similar to that observed with full-length antibody [18]. The scFv-Fc displayed on VP1/VP2-scFv-Fc VLPs not only showed VLY-binding activity but also were recognized by both anti-mouse IgG and anti- human IgG secondary antibody in ELISA confirming that all parts of the scFv-Fc molecule were properly folded and active antibody fragments were exposed on the surface of VLPs.

## Conclusions

Production of recombinant anti-VLY scFv-Fc proteins in yeast was tackled with problems of their stability, solubility and weak VLY-neutralizing activity. The addition of yeast α-factor signal sequence did not support the secretion of anti-VLY scFv-Fc protein but increased the level of its intracellular soluble form. An approach to express anti-VLY scFv-Fc molecules displayed on the surface of HaPyV-derived VLPs allowed generation of novel functionally active proteins with multivalent VLY-binding capacity, prevented scFv-Fc aggregation and improved expression. Our study demonstrated for the first time that large (472 aa long) complex protein such as recombinant antibody fused with HaPyV-derived VP2 protein and displayed on the surface of pseudotype VP1/VP2 VLPs was properly folded and exhibited a strong antigen-binding activity. Taken together, these data show that HaPyV-derived pseudotype VP1/VP2-scFv-Fc VLPs represent a successful alternative for the generation of multivalent antibody molecules in yeast *S. cerevisae *and broadens the potential of HaPyV-derived VLPs as a highly efficient carrier for functionally active complex proteins.

## Methods

### Generation of yeast expression plasmids

All DNA manipulations were carried out according to standard procedures [40]. Enzymes and kits for DNA manipulations were purchased from Thermo Scientific Fermentas (Vilnius, Lithuania). Recombinants were screened in *E. coli *DH10B cells. The yeast expression vector pFX7 for target protein expression was previously described [22]. Vector pFGG3-VP1/VP2Bg used for target protein fusion with VP2 protein was constructed by inserting DNA sequence encoding HaPyV VP1 into GAL7 expression cassette and modified HaPyV VP2 gene under GAL10-PYK1 hybrid promoter into pFGG3 plasmid [39]. The 1-100 aa encoding sequence in modified VP2 protein was deleted and GSS linker encoding sequence along with the BglII restriction site at N-terminus was introduced for fusion with target protein encoding sequence.

DNA sequences encoding the anti-VLY scFv were cloned from hybridoma 9B4 producing neutralizing MAbs against VLY [18]. The anti-VLY scFv comprising variable regions of mouse IgG heavy (VH) (GenBank JF951748), and light chains (VL) (GenBank JF951747) in the orientation of VL-linker 20 aa-VH was fused with the Fc region of human immunoglobulin G1 encoded in pFUSE-hIgG1-Fc2 vector [9]. The resulting construction obtained by fusion of anti-VLY scFv to human Fc region was named anti-VLY scFv-Fc. Four yeast expression plasmids carrying the anti-VLY scFv-Fc DNA fragment were generated.

#### Construction of pFX7-scFv-Fc plasmid

The scFv-encoding DNA fragment was amplified to introduce EcoRV and BcuI sites at the 5'-end and BglII site at the 3'-end of the PCR fragment using 5'-CGGATATCACTAGTTATGGATATTGTGATGACACAGAC and 5'-ATGAGATCTTGAGGAGACGGTGACTGAGGT primers (restriction enzyme sites underlined). The obtained PCR product was sequenced, digested with EcoRV and BglII and fused with human Fc-encoding region by cloning into EcoRV-BglII digested pFUSE-hIgG1-Fc2 vector [9]. The resulting DNA sequence encoding scFv-Fc fuse was cut out with BcuI-NheI and subcloned into the XbaI site of the yeast expression vector pFX7. The pFX7-scFv-Fc plasmid was used for the expression of non-tagged scFv-Fc construct.

#### Contruction of pFX7-His-scFv-Fc plasmid

The His-tag sequence (presented in italic) was added in-frame into the 5'-end of the scFv-encoding fragment by PCR using the oligonucleotide primers 5'-CGGATATCACTAGTTATG*CACCACCACCACCATCAC*GATATTGTGATGACACAGACTAC and 5'-ATGAGATCTTGAGGAGACGGTGACTGAGGT. The 5'-end PCR primer carries EcoRV and BglII sites (underlined) on 5'-end and 3'-end PCR primer appends BglII site (underlined) on 3'-end of the scFv-Fc for fusion with Fc-encoding region in pFUSE-hIgG1-Fc2 vector. The DNA sequence encoding His-tagged scFv-Fc was cut out with BcuI-NheI and subcloned into XbaI digested pFX7 to generate plasmid pFX7-His-scFv-Fc. The pFX7-His-scFv-Fc plasmid was used for the expression of scFv-Fc construct with His-tag at the N-terminus (His-scFv-Fc).

#### Construction of pFX7-αF-scFv-Fc plasmid

A plasmid containing yeast α-factor signal sequence was used as a template for amplification by PCR using primers containing BcuI and AarI (underlined) cleavage sites 5'-GCTGATCACTAGTTATGAGATTTCCTTCAATTTTTACTG and 5'-GCACCTGCACTCTCTTTTATCCAAAGATACCCCTTC. Respectively, AatI site was introduced into 5'-end of scFv DNA fragment by PCR and fused with α-factor signal sequence. The coding sequences for α-factor and scFv were sequenced to exclude PCR-prone errors. The BglII-MlsI digested Fc coding DNA sequence was fused with α-scFv and the resulting DNA fragment α-scFv-Fc was cloned into the XbaI site of pFX7 to generate plasmid pFX7-αF-scFv-Fc. This plasmid was used for the expression of scFv-Fc construct containing yeast α-factor signal sequence at its N-terminus (αF-scFv-Fc).

#### Construction of pFGG3-VP1/VP2-scFv-Fc plasmid

The Fc fragment-coding sequence was amplified to remove stop codon at the 3'-end and introduce XbaI and BamHI sites (underlined) at the 5-' and 3'-ends of the PCR fragment, respectively, using primers 5'-CGTCTAGACGATATCGGCCATGGTTAGATCTG and 5'-CAGGATCCGAACCTCTTGGAACCAATTTACCCGGAGACAGGGAGAGGCTC. The resulting DNA fragment verified by sequencing was cloned into the XbaI and BglII sites for fusion to modified HaPyV VP2-encoding sequence in pFGG3-VP1/VP2Bg vector. The scFv encoding fragment digested with BcuI and BglII as described above was cloned into pFGG3-VP1/VP2 plasmid carrying the sequence encoding chimeric Fc-VP2 protein. The plasmid pFGG3-VP1/VP2-scFv-Fc was used for the co-expression of chimeric VP2-scFv-Fc and VP1 proteins in yeast.

### Yeast strains, growth media and cultivation conditions

The resulting plasmids pFX7-scFv-Fc, pFX7-His-scFv-Fc, pFX7-αF-scFv-Fc and pFGG3/VP1-VP2-Fv-Fc-VP2 were used for the transformation of *S. cerevisiae *strain AH22-214 (*a, leu2-3,112, his4-519*) and its derivative AH22-214p (*a, leu2-3,112 his4-519, ura3, Δpep4*) lacking peptidase Pep4. Yeast transformants harboring plasmids with scFv-Fc encoding genes were grown in YEPD medium (yeast extract 1%, peptone 2%, and glucose 2%) supplemented with 5 mM formaldehyde overnight at 30°C and recombinant protein expression was induced after transferring yeast cells into induction medium YEPG (yeast extract 1%, peptone 2%, and galactose 3%) supplemented with 5 mM formaldehyde and culturing for additional 18 h. Yeast biomass harboring recombinant proteins was harvested by centrifugation and was stored at -20°C. Yeast transformed with vector pFX7 or pFGG3 without any insert was used as a negative control.

### SDS-PAGE and Western blotting

Proteins were analyzed by electrophoresis on 12.5% sodium dodecylsulfate-polyacrylamide gels (SDS-PAGE) followed by Coomassie brilliant blue staining. Briefly, 20-50 mg of yeast cell pellets were collected by centrifugation, washed with distilled water and suspended in 20-50 μL of DB 150 buffer (150 mM NaCl, 1 mM CaCl_2_, 0.001% Trition X-100, 0.25 M L-Arginine in 10 mM Tris/HCl-buffer, pH 7.2). An equal volume of glass beads was added and the cells were lysed by vortexing for 7 min with cooling on ice for 1 min between each vortexing step. The samples of whole yeast lysates, the supernatant and pellets obtain after centrifugation of whole yeast lysate or purified proteins were mixed with the SDS-PAGE sample buffer (Thermo Scientific Fermentas), boiled for 5 min, applied to a SDS-PAGE and run in SDS-Tris-glycine buffer. Protein bands in SDS-PAGE were visualized by staining with Coomassie brilliant blue (Sigma-Aldrich, St. Louis, MO, USA) or electro-transferred to Immobilon P membrane (Millipore, Bedford, MA, USA). The membranes were blocked with 5% milk in phosphate-buffered saline (PBS) for 2 h at room temperature (RT). Blocking solution was removed and the membranes were incubated for 1 h at RT with horseradish peroxidase (HRP) conjugated rabbit polyclonal antibodies against human IgG (Bio-Rad, Hercules, CA, USA) diluted 1:2000 in PBS with 0.1% Tween 20 (PBST) or mouse polyclonal antibodies against HaPyV VP1/VP2 VLPs generated in-house and diluted 1:1000 in PBST. For the identification of VP1/VP2-scFv-Fc protein the membrane before developing was additionally incubated with HRP-conjugated goat anti-mouse IgG (Bio-Rad) diluted 1:1000 in PBST. The enzymatic reaction was developed using tetramethylbenzidine (TMB) chromogenic substrate (Sigma-Aldrich).

### Purification of αF-scFv-Fc protein and pseudotype VLPs harboring scFv-Fc

*S. cerevisiae *yeast biomass harboring recombinant proteins, was suspended and homogenized in DB450 buffer (450 mM NaCl, 1 mM CaCl_2_, 0.001% Trition X-100, 0.25 M L-Arginine in 10 mM Tris/HCl-buffer pH 7.2) containing 2 mM PMSF, EDTA-free Complete Protease Inhibitor Cocktail (Thermo Scientific Fermentas) and mechanically disrupted using French press.

#### Purification of the αF-scFv-Fc protein

After centrifugation of yeast lysate the supernatant containing the αF-scFv-Fc protein was collected and loaded onto the protein A Sepharose column (rProtein A Sepharose Fast Flow, GE Healthcare, Sweden) equilibrated with 0.1 M Tris-HCl (pH 8.0) buffer. The column was washed with 0.1 M Tris-HCl (pH 8.0) followed by a wash step with 0.01 M Tris-HCl (pH 8.0). The elution was performed with 0.05 M Glycine-HCl (pH 3.0) buffer. Fractions were collected and the presence of the functionally-active αF-scFv-Fc protein was verified by an indirect ELISA. The fractions containing purified αF-scFv-Fc were pooled and dialyzed against PBS.

#### Purification of pseudotype VLPs harboring scFv-Fc

After centrifugation of yeast lysate, the supernatant containing VP1/VP2-scFv-Fc proteins was collected and loaded onto a 20-69% sucrose gradient. Subsequently, a centrifugation at 100,000 × g (Beckman Optima LE-80K Ultracentrifuge, Brea, CA, USA) overnight at 4°C followed. Thereafter, fractions of 0.5 mL were collected and samples were subjected to SDS-PAGE followed by Coomassie brilliant blue staining. Fractions showing a protein band with an apparent molecular mass of approximately 42 kDa corresponding to VP1 protein were pooled and diluted in DB150 buffer. The mixture was subjected to the ultracentrifugation on CsCl gradient with densities from 1.23 to 1.42 g/ml overnight at 100,000 × g (Beckman). Collected fractions were analyzed as described above. After pooling of the VP1-containing fractions, the second centrifugation in CsCl density gradient was performed. CsCl gradient fractions containing recombinant VP1/VP2-scFv-Fc proteins were diluted and precipitated by ultracentrifugation for 4 h at 100,000 × g (Beckman). The pellets were dissolved in PBS and dialyzed against PBS overnight. The dialyzed VP1/VP2-scFv-Fc proteins were aliquoted and lyophilized or stored in PBS in 50% glycerol at -20^°^C. The VLY-binding activity of the VLPs harboring the scFv-Fc fragment was verified by an indirect ELISA.

### Determination of the quantities of VP1 and VP2-scFv-Fc proteins in VP1/VP2-scFv-Fc VLPs

The concentration of purified VP1/VP2-scFv-Fc VLPs VLPs (mg/ml) was determined by Bradford assay. Known quantities of VP1/VP2-scFv-Fc VLPs (2, 2.5, 3 μg per lane) were loaded on the SDS-PAGE gel. The quantities of VP1 and VP2-scFv-Fc proteins in each VLP preparation loaded on the gel was determined by the densitometric scanning of the Coomassie brilliant blue stained protein bands fractionated by SDS-PAGE, using the ImageScanner III (GE Healthcare, Little Chalfont, UK) device. Quantitative analysis was conducted with the ImageQuantTL software supplied with the instrument. The determination of the quantities of VP1 and VP2-scFv-Fc proteins in each VLP preparation was based on the comparison with the known quantities of VP1 and BSA proteins run in the same gel. At least three scans of each VLP preparation were obtained. The determined quantities of VP1 protein (1.88, 2.23 and 2.76 μg) and VP2-scFv-Fc protein (0.19, 0.22 and 0.28 μg) in these three VLP preparations corresponded to 44.92, 53.28, 65.94 pmoles of VP1 protein and 2.38 2.76, 3.51 pmoles of VP2-scFv-Fc protein as calculated in accordance with their MW. As the number of molecules in one mole is known (Avogadro constant), the number of molecules of VP1 protein (2.70 × 10^13^, 3.21 × 10^13^, 3.97 × 10^13^) and VP2-scFv-Fc protein (1.434 × 10^12^, 1.66 × 10^12^, 2.11 × 10^12^) in three analyzed VLP preparation samples were determined. Assuming that 360 molecules of VP1 protein are needed for the assembly of one VLP [33] the number of VP1 molecules calculated above was divided by 360 to determine the number of VLPs (7.51 × 10^10^, 8.91 × 10^10^, 1.10 × 10^11^) in three analyzed samples. The number of VP2-scFv-Fc molecules presented on one VLP (19.1, 18.64, and 19.21) was calculated by dividing the number of VP2-scFv-Fc protein molecules by the number of VLPs in the sample. The determined average number of VP2-scFv-Fc protein molecules per pseudotype VLP was 19.

### Electron microscopy

The samples of purified recombinant VP1/VP2-scFv-Fc protein were placed on 400-mesh carbon coated palladium grids, negatively stained with 2% aqueous uranyl acetate solution and examined by Morgagni 268 electron microscope (FEI Inc., Hillsboro, OR, USA).

### Indirect ELISA

Polystyrene microtiter plates (Nerbe plus, Winsen/Luhe, Germany) were coated with 100 μL per well of recombinant VLY diluted in coating buffer (0.05 M sodium carbonate, pH 9.6) to a concentration of 5 μg/mL and incubated overnight at 4^°^C. The coated plates were blocked with 2% BSA added 150 μL/well for 2 h at RT. Plates were rinsed twice with PBST. Recombinant αF-scFv-Fc protein and VP1/VP2-scFv-Fc VLPs were serially diluted in PBST, added to the wells and incubated for 1 h at RT. Concentrations of αF-scFv-Fc and VP2-scFv-Fc protein displayed on VP1/VP2-scFv-Fc VLPs ranged from 150 μg/mL to 1.2 μg/mL and from 100 μg/mL to 0.8 μg/mL, respectively. The plates were rinsed 3 times with PBST and incubated for 1 h with HRP-conjugated rabbit anti-human IgG (Bio-Rad) diluted 1:1000 in PBST or HRP-conjugated goat anti-mouse IgG (Bio-Rad) diluted 1:5000 in PBST. The plates were rinsed 5 times with PBST. Enzymatic reaction was visualized by the addition of 100 μL of ready-to-use TMB substrate (Sigma-Aldrich) to each well. After 10 min of incubation at RT, the reaction was stopped by adding 50 μL/well of 10% sulphuric acid. The optical density (OD) was measured at 450 nm (reference filter 620 nm) in a microplate reader (Tecan, Groedig, Austria).

### *In vitro *hemolytic assay using human erythrocytes

In vitro hemolytic assay was performed as described previously [18]. Briefly, human blood specimens were collected by a venipuncture from healthy adult volunteer and anticoagulated by EDTA. Erythrocytes were isolated by centrifugation and resuspended in sterile PBS. For the VLY neutralization assay, VLY (5 ng/mL) was preincubated with serial dilutions of recombinant purified αF-sFv-Fc protein and VP1/VP2-scFv-Fc VLPs for 30 min at 20°C. Concentrations of the αF-scFv-Fc ranged from 3.6 ng/mL to 0.54 μg/mL (6 × 10^-11 ^M to 8.96 x10^-9^). Calculated concentrations of VP2-scFv-Fc on pseudotype VP1/VP2-scFv-Fc VLPs ranged from 1 ng/mL to 33 ng/mL or 1.25 x10^-11 ^M to 4.1 × 10^-10 ^M. The obtained mixture was added to 1% erythrocyte suspension in PBS. As a negative control, recombinant VLY was added to 1 mL of 1% erythrocyte suspension in PBS to the final concentration of 5 ng/mL. As a positive control, VLY pre-incubated with the full-length neutralizing mAb 9B4 was used. The concentration of the MAb 9B4 ranged from 1 ng/ml (6.7 × 10^-11 ^M) to 200 ng/ml (1.33 × 10^-9 ^M). After 15 min of incubation at RT the cells were pelleted by centrifugation and the released hemoglobin was measured at 415 nm wave length (OD415) in a microplate reader (Tecan).

### Cytotoxicity assay using HeLa cell line

Adherent human epitheloid HeLa cells (ATCC Cat. No. CCL-2) were cultivated in RPMI-1640 growth medium (Biochrom, Berlin, Germany) supplemented with 10% fetal bovine serum (Biochrom) and antibiotics. The cells were grown at 37°C and 5% CO_2 _in 96-well plates to approximately 70% confluence. After removing growth medium, the cell monolayer was rinsed twice with serum-free RPMI-1640 medium and then serum-free RPMI-1640 medium was added to the cells (50 μL/well). Recombinant VLY was diluted in serum-free RPMI medium and preincubated with serial dilutions of VP1/VP2-scFv-Fc VLPs for 30 min at RT. The concentration of VLY in each incubation mixture was 6 μg/mL, the concentrations VP2-scFv-Fc displayed on VP1/VP2-scFv-Fc VLPs ranged from 0.14 to 10 μg/mL. Fifty microliters of each mixture were added to the wells with HeLa cells and the plates were incubated for 1 h at 37°C and 5% CO_2_. Thus, after adding the incubation mixture (50 μL/well) to the culture of HeLa cells (50 μL/well) the final concentration of VLY in each well was 3 μg/mL. As a negative control, recombinant VLY at the final concentration of 3 μg/mL was used. As a positive control, VLY pre-incubated with the full-length neutralizing mAb 9B4 (50 μg/mL) was used. After incubation, cell viability was determined by a colorimetric assay using 3-(4,5-dimethylthiazol-2-yl)-5-(3-carboxymethoxyphenyl)-2-(4-sulfophenyl)-2H-tetrazolium (MTS) staining. Fifty microliters of ready-to-use MTS solution (Promega, Madison, WI, USA) were added to the wells and the plates were incubated for 1 h at 37°C and 5% CO_2_. The OD was measured at 490/630 nm wave length in a microplate reader (Tecan). Cell viability was also assessed microscopically at magnifications 20x and 40x using microscope Olympus IX-70 (Olympus, Japan).

## List of abbreviations

aa: amino acid; CDC: cholesterol-dependent cytolysin; HaPyV: hamster polyomavirus; HaPyV VP1 and VP2: hamster polyomavirus capsid proteins VP1 and VP2; HRP: horse-radish peroxidase; IgG: immunoglobulin G; PBS: phosphate buffered saline; scFv: single-chain variable fragment of immunoglobulins; scFv-Fc: single-chain variable fragments of immunoglobulins fused with Fc domain of human IgG1; SDS-PAGE: sodium dodecyl sulfate polyacrylamide gel electrophoresis; VH: variable region of immunoglobulin heavy chain; VL: variable region of immunoglobulin light chain; VLY: vaginolysin; VLP: virus-like particle.

## Competing interests

The authors declare that they have no competing interests.

## Authors' contributions

MP was involved in all aspects of the experimental design, construction of plasmids, purification of pseudotype VLPs, data collection, analysis and interpretation and the manuscript drafting. AZ set up the cytolytic assay, drafted and edited the manuscript. IS purified the recombinant proteins and carried out the immunoassay. AG conceived of the study, was involved in all aspects of the experimental design, analysis and interpretation, drafted and edited the manuscript. All authors contributed to the final version of the manuscript.
